# Ginger compress therapy for adults with osteoarthritis

**DOI:** 10.1111/j.1365-2648.2010.05355.x

**Published:** 2010-10

**Authors:** Tessa Therkleson

**Affiliations:** Tessa Therkleson PhD RN Director of Nursing RATO HealthLower Hutt, New Zealand

**Keywords:** ginger compress therapy, Giorgi’s method, nursing, osteoarthritis, phenomenology

## Abstract

therkleson t. (2010) Ginger compress therapy for adults with osteoarthritis. *Journal of Advanced Nursing***66**(10), 2225–2233.

**Aim:**

This paper is a report of a study to explicate the phenomenon of ginger compresses for people with osteoarthritis.

**Background:**

Osteoarthritis is claimed to be the leading cause of musculoskeletal pain and disability in Western society. Management ideally combines non-pharmacological strategies, including complementary therapies and pain-relieving medication. Ginger has been applied externally for over a thousand years in China to manage arthritis symptoms.

**Method:**

Husserlian phenomenological methodology was used and the data were collected in 2007. Ten purposively selected adults who had suffered osteoarthritis for at least a year kept daily diaries and made drawings, and follow-up interviews and telephone conversations were conducted.

**Findings:**

Seven themes were identified in the data: (1) Meditative-like stillness and relaxation of thoughts; (2) Constant penetrating warmth throughout the body; (3) Positive change in outlook; (4) Increased energy and interest in the world; (5) Deeply relaxed state that progressed to a gradual shift in pain and increased interest in others; (6) Increased suppleness within the body and (7) More comfortable, flexible joint mobility. The essential experience of ginger compresses exposed the unique qualities of heat, stimulation, anti-inflammation and analgesia.

**Conclusion:**

Nurses could consider this therapy as part of a holistic treatment for people with osteoarthritis symptoms. Controlled research is needed with larger numbers of older people to explore further the effects of the ginger compress therapy.

**What is already known about this topic**

- Osteoarthritis is the most common form of musculoskeletal pain and disability in older adults in Western society.
- People with osteoarthritis suffer a variety of symptoms, including pain, psychological distress, social isolation and general inability to cope.
- Management of osteoarthritis ideally involves both conventional pain-relieving medication and non-pharmacologic strategies, such as ginger compress therapy.

**What this paper adds**

- The essential experience of ginger compresses exposed the unique qualities of heat, stimulation, anti-inflammation and analgesia.
- Seven interpretive themes of the experience of a series of ginger kidney compresses were identified.
- From these seven themes, three essential themes were identified which summarize the phenomenon of the ginger kidney compress: warmth penetrated through the entire self, activating deep relaxation, total relaxation of the self enabled release of tension and improved receptivity towards others. Additionally, interest in the outer world increased as the self felt more mobile and energized.

**Implications for practice and/or policy**

- Ginger compress therapy should be considered as a viable non-invasive option by nurses caring for people with osteoarthritis.
- Further controlled research on ginger compress therapy is needed with larger numbers of the elderly suffering from osteoarthritis.

## Introduction

Osteoarthritis (referred to as OA) is a chronic, degenerative arthritis which typically results in a thinning of joint cartilage in the knees, hips, spine and/or hands (Grainger & Cicuttini 2004). It is the most prevalent cause of musculoskeletal pain and disability in older people in Western cultures (Warsi *et al.* 2003, Rahman 2005), and its management ideally involves a multidisciplinary approach (Felson *et al.* 2000b). Ginger compresses are used to manage arthritis symptoms in Chinese folk medicine (Xinangcai 1998) and European hospitals specializing in complementary healthcare (Eichler 1981, Fingado 2001).

## Background

The World Health Organization declared 2000–2010 the Bone and Joint Decade, with one of its primary aims being the advancement of research and management of musculoskeletal disorders such as OA. Whereas OA primarily affects older people, with at least 80% of those over the age of 65 years having radiographic evidence of OA, fewer than 25% are symptomatic (Felson *et al.* 2000a, Rahman 2005). X-rays sometimes show evidence of OA when there are no symptoms and, conversely, OA symptoms may occur when there is little radiographic evidence of OA (Felson *et al.* 2000a). OA is a complex disease and joint degeneration, as observed by X-ray, results in varying degrees of pain and immobility, with other factors such as quality of life and psychosocial issues having an impact on the people with this condition (Rosemann *et al.* 2006).

Osteoarthritis management is conventionally concerned with controlling symptoms of pain and lack of mobility through the use of non-steroidal anti-inflammatory drugs (NSAIDS) and analgesics (Hunt *et al.* 2009). These conventional medications are often rejected by people, either because of side effects from long-term use or personal preference (Felson *et al.* 2000b, Fendrick & Greenberg 2009). Rheumatologists propose that management ideally combines conventional pain-relieving medication with non-pharmacological strategies, such as changes in diet, exercise and the use of natural therapies (Felson *et al.* 2000b). Research shows that people with OA respond positively to heat therapy (Flusser *et al.* 2002, Cantarini *et al.* 2007) and relaxation therapy (Gay *et al.* 2002, Baird & Sands 2006). The ginger compress, when applied over the kidney region, combines both heat and relaxation therapies, with the addition of an awakening and stimulating effect (Schurholz *et al.* 1992/2002, Therkleson & Sherwood 2004). Schurholz *et al.* (1992/2002) reported that the ginger kidney compress was especially helpful for arthritis. Researchers have claimed that oral ginger extract can be used to manage the symptoms of arthritis, with human random controlled trials showing a statistically significant reduction of OA symptoms (Bliddal *et al.* 2000, Altman & Marcussen 2001, Haghighi *et al.* 2005). However, these studies report that the high dose of oral ginger extract required to achieve the desired response has often led to gastrointestinal complaints (Marcus & Suarez-Almazor 2001).

Limited research has been conducted on ginger applied externally, alhough it has been applied to painful joints for at least a thousand years in ancient Chinese folk medicine (Xinangcai 1998). Schurholz *et al.* (1992/2002) in Germany carried out a pilot study in 1990 with over 300 nurses and doctors, followed by a comprehensive study between 1991 and 1992, when 800 ginger compresses were applied over the kidney region to 41 patients with a variety of health conditions. The ginger kidney compress (GKC) was found to be especially helpful in cases of arthritis, kidney stones, bronchitis, asthma, pneumonia and some forms of depression. A phenomenological study based in one city in New Zealand identified a positive experience for seven people with different health conditions receiving one GKC in private nursing clinics (Therkleson & Sherwood 2004). In neither of these studies was the phenomenon of the GKC as reported by people with OA examined.

## The study

### Aim

The aim of the study was to explicate the phenomenon of ginger compresses for people with osteoarthritis.

### Methodology

A Husserlian phenomenological approach was selected. The phenomenological approach considers human experience as its data and uses a defined process to explore these data and expose the phenomenon, that is, what is understood from the perspective of the experiencing participant (Giorgi 2000). The phenomenon in this study was the GKC, and its meaning was described by ten participants in words, both verbal and written, and pictures.

Giorgi (2008) emphasizes the importance of using phenomenological reduction, which includes imaginative variation and bracketing, to expose the meaning of a phenomenon from the data. Phenomenological reduction is an attitude of mind applied in the understanding of the data to enable the researcher to observe and identify the phenomenon being experienced free of personal biases. Bracketing is a form of self-reflection that places the objective world in parenthesis and suspends personal biases, beliefs and opinions in order to explore the phenomenon in its totality (Husserl 1983). Throughout the research process, a detailed reflective journal was used as an aid to bracketing. This journal allowed critical consciousness to enter the research process and to direct ideas back to the research practice. Throughout the study, a professional mentor provided personal support for the researcher, and two academic supervisors assisted in the design and explication process.

### Participants

A sample of ten consenting adults over 45 years, who had been diagnosed with symptomatic osteoarthritis for at least 1 year, was purposively selected. Osteoarthritis was confirmed by X-ray and completion of the Short Arthritis Assessment Scale (Wolfe *et al.* 2004). The health assessment excluded rheumatoid arthritis, fibromyalgia, cancer and other serious conditions, and those taking corticosteroids. In phenomenological research the sample size must be sufficient to understand the phenomenon (Giorgi 1997). Ten participants ensured that an in-depth profile of the experience was gained that represented the core themes and enabled understanding of the phenomenon of the GKC.

### Application of the ginger kidney compress

The participants were offered the GKC by doctors and nurses in five separate primary healthcare clinics in New Zealand and Australia. The nurses applied the GKC daily for seven consecutive days according to a defined procedure and protocol as clarified by Schurholz *et al.* (1992/2002). The GKC comprised a cotton cloth soaked in a hot ginger infusion and applied for 30 minutes over the kidney region, followed by a 20-minute rest. During the GKC, participants rested supine in a comfortably warm and quiet place.

### Data collection

The data were collected over a 9-month period in 2007 from four sources: (1) written diaries daily describing the GKC experience; (2) coloured human diagrams on which participants indicated warmth as red, cold as blue and sensations as yellow; (3) interviews within one week of the treatment; and (4) two follow-up telephone conversations after the interview. The primary question asked in the diaries and interviews was the same for all participants: ‘What was your experience of the GKC?’ During the interviews, further supplementary questions were used as prompts, such as: ‘Could you tell me more about that?’ and ‘Could you describe how that made you feel?’ The in-depth, open-ended interviews conducted 1 week after the treatment took 40–80 minutes and took place in participants’ own homes. The data obtained were rich and varied. As Giorgi (1997) claims, written and coloured descriptions are brief and organized, whereas interviews are generous, disorganized and spontaneous.

### Ethical considerations

The study was approved by the appropriate ethics committees. Ten Registered Nurses, educated in ginger compress therapy, delivered the treatment and monitored participants’ condition.

### Data explication

Explication of the data was systematic and followed six clearly defined steps as adapted by Schweitzer (1983, 1998) from Giorgi (1971, 1985, 1997). These steps were: (1) holistic grasp of all the data; (2) delineation of data into discrete meaning units in the participants’ language, with the development of individual participant profiles; (3) amalgamation and coding of all participant data; (4) interrogation of the amalgamated coded data and consideration of emerging themes in the researcher’s language; (5) description and discussion of themes; and (6) succinct summary of themes to isolate the phenomenon of the GKC for people with OA. The software tool, Nvivo 7 (Richards 2005, 2006), was used to facilitate management and analysis of the data.

### Rigour

A phenomenological stance was adopted, which involved transparent bracketing and a systematic, explicit process in making decisions throughout the research process. Data were obtained in a variety of ways and were visible and accessible to others. The use of Nvivo 7 software enabled memos of thought processes and creative imaginings to be recorded alongside the analysis. In analysing the data, experienced Husserlian phenomenologists were involved in offering critique and guidance.

## Findings

Seven themes were identified, representing the core experience for the participants. For all participants except one, the changes were present for at least a month following the treatment. This one exception was conscious of the changes during each GKC yet suffered extreme pain, which necessitated opiate medication and a joint replacement within 4 weeks of the treatment.

### Theme 1: Meditative-like stillness and relaxation of thoughts

Participants experienced warmth building up in the body, which initially spread from the mid-point of the back to the head, activating a sense of meditative-like stillness and relaxation of thoughts. This changed mental state allowed the opportunity to positively reconsider challenges in life, eventually arousing an inner state of peace and calm.

This theme included the features of warmth, relaxation, peace, calm and comfort in association with participants’ thought processes. In a warm, comfortable and semi-dozing place, they reassessed and released mental worries and tensions, which led to overall calm and composure:

I am sure many times when the nurse came in I just didn’t want to come back into the world, you know, you just wanted to stay there where your body was so lovely and comfy; it was perfect.

Picturesque words were used to describe the somewhat dreamy experience:

It was cosy and warm and I felt like I was floating on a cloud.

Prior to the treatment, there had been a tendency to hurry and panic through life, whereas during the GKC a space of tranquillity replaced that of general tension:

From the rushing in and everything being so outwardly alert and living on nerves, I came to a much more peaceful, relaxing situation.

In this peaceful place, while totally present, the mind experienced a profound state of stillness. Issues in life that had been disturbing were met with inner serenity. In warmth and comfort, OA no more featured and life could be considered with renewed understanding.

### Theme 2: Constant penetrating body warmth throughout the body

Participants experienced constant penetrating warmth, which gradually increased in intensity and radiated throughout the body, from the mid-back to the head then extending to the feet, hands and inner body, activating an overall physical warmth and relaxation. The second theme amalgamated the features of warmth and relaxation.

Following every GKC, participant’s coloured warmth on a human diagram using red, which intensified on the back and extended to include the feet, hands and OA joints. Participants initially indicated cold feet and OA joints by colouring them blue and/or describing this:

I haven’t experienced that warmth before, you sort of feel penetrating warmth that was getting into your bones; it wasn’t surface warmth. You know how you stand in front of a fire and get warm and as soon as you move away it’s gone, well this is constant.

Heat and warmth flowed through the body, extending out to the periphery:

It did just seem to spread; my back got warm then it was sort of as if my whole nervous system was saying, ‘This is really nice, I’ll have some as well’. It spread down both legs and arms, that lovely warmth was so good.

Warmth increased in the joints and the metabolic and excretory organs, with a corresponding positive effect on excretion and digestion. Depending on the participant’s constitution, the warmth seemed to be directed to where it was most beneficial and was perceived as activated by the ginger, with words such as hot, spicy, prickly, itchy, tingly, glowing and fuzzy used. The increased bodily warmth led to a sense of deep relaxation, allowing release of bodily tensions:

You are relaxed and if you are relaxed it doesn’t matter what you are doing you just get on with it and don’t think about it. If you are in pain and you can’t get up from sitting down or anything the brain takes over and tells you there is an obstruction. I felt a greater freedom once I got off the table and was warmed up.

Whilst these reports were subjective, a common picture of freedom and positivity emerged as the OA obstruction shifted.

### Theme 3: Positive change in outlook

Participants experienced a positive shift in thinking, with a subsequent change in outlook. Past memories, of family, friends and health were awoken and met with fresh insight and acceptance, leading to renewed interest and confidence in relationships with others.

During the GKC there was a warming and loosening of thought processes. Consciousness shifted from the present to the past and on into the future, with an internal reliving of experiences with others:

Thoughts at the start were all the external things that I have to think about in the day; these quickly disappeared. I could align my thoughts in whatever way I wanted to in daydreaming.

Participant musings after the GKCs revealed an underlying regret for losses of the past, such as physical agility, nurture and relationships with family and friends. Living with OA was frustrating and caused loss of freedom:

I experienced OA as a disability; like having to get by with a broken part, yet 7 years ago I could hardly keep my feet on the ground and I would run everywhere.

As participants reminisced, they became increasingly aware of the significance of others, with an inner ordering and shift in understanding:

My father died at my age, crippled with OA; in fact my mother became confused and thought I was her husband at one stage.

Perceptions changed as memories from the past were awoken in the present. Thinking that had been closed, even limited, became clearer and more outward looking:

Some of my family members, they probably didn’t know the extent of my disability. Because there is nothing much you can do about it if you keep going on about it. I wasn’t actually hiding it; I was just not sharing with anyone. Now I have done a lot of talking to friends and people I have met.

Following the treatment, participants described a change from being reserved and reticent to having more meaningful social interaction, with increased interest in how others managed a disability such as OA.

### Theme 4: Increased energy and interest in the world

Following the GKC, participants felt awake and alive, arousing both new and rejuvenated interests in worldly activities.

Increased focus at work and home meant that no more were thoughts being dissipated; rather, thinking was more alive:

This week I feel things are flowing, I am going back to a creative style that I thought I had lost in my work; there is more movement in my head and thinking.

Whilst receiving daily GKCs meant a busy week for participants, there was no mention of fatigue; rather, life took on a fresh vigour:

After the compress I wake up feeling awesome, awake and alive. I get on with the day and find my thinking is wide awake. It’s like a buzz and I am off to work sparking.

Participants’ lightening in thinking and increase in energy stimulated more openness towards the world. Energized, focused mental processes enabled tasks, such as completing a meal, class lesson or creative artwork, to be accomplished in a more organized fashion. During and following the treatment, participants began to consider new ideas, such as travelling out of town, walking up hills and subtly changing work processes. After the GKCs, there were suggestions of personal growth, with words such as spring, butterfly, freedom and release being used:

After the week’s course I spread my wings free and remained flying for about 4 days. I still have increased energy in my thinking and I feel positive.

Participants’ thinking that was previously confused was, after the treatment, attended with enthusiasm and creativity. An attitude of positivity and hope was evident as they made plans into the future.

### Theme 5: Deeply relaxed state that progressed to a gradual shift in pain and increased interest in others

Participants experienced a gradual shift of pain as the inner body progressively warmed and relaxed, allowing a sense of emotional freedom which resulted in a new-found willingness to share their private world with family, friends and colleagues.

During the first GKCs, low-level physical irritations and discomforts were accentuated, with old injuries surfacing:

Every day as I lay down the right side of the back became quite tense. I didn’t have a sore back when I lay down; it was like the experience of an old pain or tension.

The warmth of the GKC infiltrated and penetrated the body, activating a sense of opening and relaxing, with a gradual release of tension and pain:

It’s the warmth that goes through the body from those compresses that causes the relaxation. Whatever the ginger does from then on, when you are relaxed it is able to do its thing.

The extreme chronic pain shifted during the later GKCs as participants relaxed totally:

I was totally relaxed with the GKC; a time free of pain, great.

They contemplated the changed experience and described an association between warmth, relaxation and movement.

As they warmed and relaxed there was an overall release from discomforts of the past – physical, mental and emotional. Following the treatment, a new attitude of optimism, even playfulness, was evident as participants smiled and joked. With a twinkle in his eye, the oldest participant said:

I came home and my wife ran a mile – not really!

Socially, there was a loosening in relating to others, with increased willingness to share inner needs:

Now I just say ‘yes please’. I haven’t been good at asking for help and accepting the fact that people are now opening doors for me.

Participants expressed an overall change in emotional being as they related to others with a new-found sense of openness.

### Theme 6: Increased suppleness within the body

Participants experienced increased suppleness in the body, which facilitated mobility, posture and breathing and positively influenced social opportunities.

Mobility was the most frequently discussed aspect of the OA experience. Initially, movement was difficult, especially where the OA joint was involved. Participants had a tendency to distance themselves from the OA experience, with subsequent loss of body awareness:

I am not inclined to be self aware of my foot and stuff like that; people will see me limping around but I am so busy I am not aware whether it is hurting or whether I am cold or hot, basically I am just too busy to be aware.

During the GKCs, the physical body became suppler as tense, contracted muscles and joints relaxed and loosened:

My whole leg is freer, I can get in and out of the car easily, I don’t have to lift my leg. When I get out of bed I can walk straight away without a pause and I think I am beginning to stand more evenly.

The increased flexibility was also evident in improved posture and more comfortable breathing:

Now I am standing up straight without even thinking about it. My posture has improved definitely. Also I can’t get over that breathing last night, I thought my breathing was so easy; I took a deep breath in and out and found it was a real flow, where before it was very shallow.

Increased mobility enabled improved interaction with others, such as visits to family and friends:

*By the end of the second compress I did think something was happening. I walked up the road to my friend. It doesn’t sound far three doors up the road– but when you have a problem like this it is like a mile.*

During the GKCs, as mobility and posture improved, the sense of self in relation to others changed and social life became re-enlivened. Previous social isolation was replaced with enthusiasm to contact and/or visit others.

### Theme 7: More comfortable and flexible joint mobility

Participants experienced more comfortable and flexible joint mobility and increased physical energy, which enabled renewed participation in worldly activities.

This theme amalgamated the features of relaxation, freedom and mobility. During the treatment, warmth and relaxation increased their mobility:

I felt the warmth in my hands and there was more movement in the hands; the warmth sort of feeds through the arms.

There was a definite consciousness that the warmth spreading and penetrating the inner body eased the joints affected by OA:

Having warmed up you are relaxed and moving freer; there is no question about that.

The scope of what was possible increased as participants attempted a wider range of activities. Previous restrictions had influenced feelings of self-esteem and independence, but this changed as many former tasks were attempted:

There are certain movements in a cultural performance that means I have to swivel and now I am swivelling without even thinking about it, which I couldn’t do before. I have more flexibility and mobility in my hip.

Participants were aware of a boost in energy, which was manifested initially in the desire to mobilize after each GKC and continued to carrying out increasing activities through the day:

I felt as though I was slightly rejuvenated, with more energy; this continued through to going to bed.

During and after the treatment, the increased energy and renewed joint mobility allowed involvement in former enjoyable recreational activities, such as gardening, travelling and walking.

### Meaning of ginger compress

These seven interpretive themes of the experience of a series of GKCs were further explored to expose the meaning of the GKC for people with OA. Three essential themes were identified, which summarize the phenomenon of the GKC:

1. Warmth penetrated through the entire self, activating deep relaxation.
2. Total relaxation of the self enabled release of tension and improved receptivity towards others.
3. Interest in the outer world increased as the self felt more mobile and energized.

The findings revealed that during the GKCs people with OA experienced prolonged, concentrated warmth on the lumbar region, and the opportunity to rest in a warm, quiet, comfortable place that allowed them to be totally present, while their bodily tensions diminished. Ginger’s unique qualities of heat, stimulation, vitality, anti-inflammation and analgesia aided the participants in this moment, activating a temporary detachment from the stress and pain of living with OA and allowing an opening out towards the world and others.

## Discussion

### Study limitations

This study exposed the essence of the phenomenon of the GKC for people with OA by exploring the subjective experiences of ten participants. Whilst ten participants ensured that an in-depth profile of the experience was gained, any conclusions drawn are too specific to be generalizable. This study was not intended to support statistically based generalizations, and further research with more participants is needed. Additionally, only participants from New Zealand and Australia were included. Whilst no core differences were found between these two cultures, further research in these and other cultures would be worthwhile.

### Experiences of ginger kidney compresses

The participants, as others with OA, suffered psychological distress, cognitive impairment, social isolation and a general inability to cope. Two phenomenological researchers have described the experience of living with OA of the knee: one found a sense of lost control and independence as a result of the constant disability (Keysor *et al.* 1998), while the other described a sense of learned helplessness, with a loss of self confidence, past physical agility and social integration (Maly & Krupa 2007). Three trials (Blouin *et al.* 2003, Mouchnino *et al.* 2005, Dohrenbusch *et al.* 2008) have shown that chronic pain in patients with OA resulted in disturbances in cognitive function and psychological well-being.

The present participants’ experiences suggest an association of the GKC with relaxation and heat therapies. Research on heat and relaxation therapies has shown positive effects in the management OA symptoms. RCTs using topical compresses of hot therapeutic mud on joints affected by OA have been found effective at Italian spa clinics (Flusser *et al.* 2002, Cantarini *et al.* 2007, Odabasi *et al.* 2008). Verhagen *et al.* (2007), in a systematic review of seven RCTs for patients with OA receiving spa therapy, suggested this as an effective treatment for OA symptoms. Deep relaxation therapies, such as hypnotic relaxation and mindfulness meditation, have been used effectively to manage self-perception of OA symptoms (Gay *et al.* 2002, Baird & Sands 2006). Morone and Greco (2007) is a structured review of relaxation therapies that reported their positive effect in the management of OA pain and discomfort.

The present findings suggest that participants’ experience of OA was typical, and that their responses to the GKC were comparable to those of people with OA receiving deep relaxation and heat therapies. The findings extend understanding of the GKC for people with OA and suggest possibilities for future treatment and management of this condition.

The following recommendations are made in relation to the three essential themes:

1. Given that warmth penetrated through the entire self, activating deep relaxation, the GKC is recommended as a non-invasive form of thermotherapy for OA. The GKC is especially beneficial for those with cold limbs and/or sensitivity to the cold.
2. Given that total relaxation of the self (mental, physical and emotional) enabled release of tensions and improved receptivity towards others, the GKC is recommended for people who tend to be intellectually preoccupied, fixed in habits, anxious, depressed, introspective, socially restrained, and/or tend to stiffen, tense, contract and/or hold their breath when moving.
3. Given that interest in the outer world increased as the person felt more mobile and energetic, the GKC is suggested for those with OA, who are tired and lack the stamina to be involved in outside activities.

Research involving well-designed controlled studies or trials is warranted. Possibilities for future research on this topic are vast as the GKC treatment is largely unexplored. For example, it is possible that the chemical components of ginger actively contribute to the warmth, relaxation, stimulatory, anti-inflammatory and analgesic effect. Research using transdermal delivery of ginger, in combination with heat packs, might improve management of OA. Research exploring the impact of the GKC on larger populations of older people with OA, especially those with mental health issues, would be valuable. Additionally, increased understanding of the characteristics of older people with asymptomatic OA could help to identify the characteristics and/or coping skills that people develop to manage the damage to joints caused by OA.

Conventional management of OA using anti-inflammatory drugs and analgesics is often an unsatisfactory answer due to side effects and/or patient compliance issues, whereas the GKC treatment offers hope and a brighter future for those living with OA. Healthcare providers should consider the GKC as part of holistic treatment for people with OA symptoms.
